# Supplementary material for: Isoleucine gate blocks K+ conduction in C-type inactivation
Source: eLife. 2024 Nov 12;13:e97696. doi: 10.7554/eLife.97696 (PMC11649237; doi:10.7554/eLife.97696)
Supplement: Supplementary file 1. [file elife-97696-supp1.docx]

| **Simulation** | **Force Field** | **Voltage (mV)** | **Solution (mM)** | **Mutation** | **Restraint (kcal/mol)*** | **Time scale (μs)** |
| --- | --- | --- | --- | --- | --- | --- |
| **1** | AMBER | +200 | 150 mM KCl | W362F, S367T,V377T | 0 | 10.0 |
| **2** | AMBER* | +200 | 150 mM KCl | W362F, S367T,V377T | 1 | 10.0 |
| **3** | AMBER | +200 | 150 mM KCl | W362F, S367T,V377T,I398N | 0 | 10.0 |
| **4** | CHARMM36m | +200 | 150 mM KCl | W362F, S367T,V377T | 0 | 10.0 |
| **5** | CHARMM36m | +200 | 150 mM KCl | W362F, S367T,V377T,I398N | 0 | 10.0 |
| **6** | CHARMM36m-NBFIX | +200 | 150 mM KCl | W362F, S367T,V377T | 0 | 10.0 |
| **7** | AMBER | +200 | 150 mM NaCl | W362F, S367T,V377T | 0 | 10.0 |
| **8** | CHARMM36m | +200 | 150 mM NaCl | W362F, S367T,V377T | 0 | 10.0 |

*Harmonic restraints were applied to C_β_ atoms of I398 to keep the isoleucine gate in the open state
