# Supplementary material for: Isoleucine gate blocks K+ conduction in C-type inactivation
Source: eLife. 2024 Nov 12;13:e97696. doi: 10.7554/eLife.97696 (PMC11649237; doi:10.7554/eLife.97696)
Supplement: Supplementary file 2. [file elife-97696-supp2.docx]

| **Interactions^#^** | **Atom Type1** | **Atom Type 2** | **E_min_ (kcal/mol)** | **R_min_ (Å)** | **E (kcal/mol)** | **R (Å)** |
| --- | --- | --- | --- | --- | --- | --- |
| Potassium-Water | POT | OT | -0.015033 | 4.211 | -17.800 | 2.67 |
| Potassium-Carbonyl | POT | O | -0.455556 | 3.044 | -26.000 | 2.48 |
| Water-Carbonyl | OT | O | -0.338333 | 3.200 | -8.950 | 1.71 |

^#^**E** and **R** are the minimized energy and distance for ion-water, ion-carbonyl and water-carbonyl interactions in the CHARMM36m-NBFIX force field, with Lennard-Jones (LJ) parameters **E_min_** and **R_min_**. Consistent with a AMBER-like force field, note that the ion-protein interaction has the most negative minimized energy.
