# Supplementary material for: Isoleucine gate blocks K+ conduction in C-type inactivation
Source: eLife. 2024 Nov 12;13:e97696. doi: 10.7554/eLife.97696 (PMC11649237; doi:10.7554/eLife.97696)
Supplement: Supplementary file 3. [file elife-97696-supp3.docx]

| **Simulation** | **Force Field*** | **Voltage (mV)** | **Conduction events (#)** | **Time scale (μs)** | **Single-channel conductance (pS)** |
| --- | --- | --- | --- | --- | --- |
| **1** | AMBER | +300 | 96 | 1.0 | 51.20 |
| **2** | CHARMM36m | +300 | 52 | 1.0 | 27.73 |
| **3** | CHARMM36m-NBFIX | +300 | 120 | 1.0 | 64.00 |
| **Combined number of conduction events** | | | 268 | 3.0 | 48.24 |

^*^Simulations are restrained to keep the intracellular gate open and the selectivity filter conductive. Estimates were calculated over the last 1μs of simulation time.
