# Supplementary material for: Isoleucine gate blocks K+ conduction in C-type inactivation
Source: eLife. 2024 Nov 12;13:e97696. doi: 10.7554/eLife.97696 (PMC11649237; doi:10.7554/eLife.97696)
Supplement: Supplementary file 4. [file elife-97696-supp4.docx]

| **Simulation** | **Force Field** | **Voltage (mV)** | **Conduction events (#)** | **Time scale (μs)** | **Single-channel conductance (pS)** |
| --- | --- | --- | --- | --- | --- |
| **2** | AMBER* | +200 | 2 | 10.0 | 0.16 |
| **4** | CHARMM36m | +200 | 40 | 10.0 | 3.20 |
| **6** | CHARMM36m-NBFIX | +200 | 2 | 10.0 | 0.16 |
| **Combined number of conduction events** | | | 44 | 30.0 | 1.17 |

^*^Simulation is restrained to keep I398 open.
