# Supplementary material for: Isoleucine gate blocks K+ conduction in C-type inactivation
Source: eLife. 2024 Nov 12;13:e97696. doi: 10.7554/eLife.97696 (PMC11649237; doi:10.7554/eLife.97696)
Supplement: Supplementary file 5. [file elife-97696-supp5.docx]

| **Channel** | **Mutations** | **ᐃG_LP_^1^ (kcal/mol)** | **ᐃE_LP_^1^ (kcal/mol)** | **ᐃG_LP_^2^ (kcal/mol)** | **ᐃE_LP_^2^ (kcal/mol)** | **ᐃᐃG^*^ (kcal/mol)** |
| --- | --- | --- | --- | --- | --- | --- |
| kv1.2-kv2.1 | wild-type | -13.43 ± 2.67 | -14.65 ± 1.01 | 6.77 ± 3.93 | -13.93 ± 1.43 | -20.93 ± 4.44 |
| kv1.2-kv2.1-2m | S367T/V377T | -4.06 ± 4.09 | -14.66 ± 1.20 | 4.72 ± 5.58 | -13.41 ± 1.20 | -10.03 ± 6.45 |
| kv1.2-kv2.1-3m | W362F/S367T/V377T | -5.02 ± 3.55 | -15.28 ± 1.32 | 6.25 ± 5.61 | -14.66 ± 1.64 | -11.90 ± 5.77 |

*ᐃᐃG = (ᐃG_LP_^1^+ᐃE_LP_^1^) - (ᐃG_LP_^2^+ᐃE_LP_^2^) is the net free-energy difference of CTX binding to the conductive (1) and dilated (2) conformations of the pore domain. Each free-energy estimate and statistical error was determined based on at least 10 independent docking solutions best describing the x-ray bound state of CTX.
