# Supplementary material for: Isoleucine gate blocks K+ conduction in C-type inactivation
Source: eLife. 2024 Nov 12;13:e97696. doi: 10.7554/eLife.97696 (PMC11649237; doi:10.7554/eLife.97696)
Supplement: Supplementary file 6. [file elife-97696-supp6.docx]

| **Force Field^#^** | **Gate** | **C_β_-C_β_ distance (Å)** | **Pore radius (Å)** | **Water density (Å^-1^)** |
| --- | --- | --- | --- | --- |
| AMBER | I398 | 12.76 ± 2.97 | 0.95 ± 0.20 | 7.03 x 10^-3^ |
|  | I398N | 10.70 ± 1.00 | 2.13 ± 0.47 | 1.33 x 10^-2^ |
| CHARMM36m | I398 | 15.21 ± 1.35 | 3.10 ± 0.33 | 1.20 x 10^-2^ |
|  | I398N | 12.04 ± 1.10 | 2.98 ± 0.57 | 1.95 x 10^-2^ |

^#^Average properties were determined over 10μs of simulation.
